# Screening for Depression in Younger Breast Cancer Survivors: Outcomes From Use of the 9-item Patient Health Questionnaire

**DOI:** 10.1093/jncics/pkab017

**Published:** 2021-02-08

**Authors:** Patricia A Ganz, Julienne E Bower, Ann H Partridge, Antonio C Wolff, Elissa D Thorner, Hadine Joffe, Michael R Irwin, Laura Petersen, Catherine M Crespi

**Affiliations:** 1 Department of Health Policy and Management, UCLA Fielding School of Public Health, Los Angeles, CA, USA; 2 Department of Medicine (Hematology-Oncology), David Geffen School of Medicine at UCLA, Los Angeles, CA, USA; 3 UCLA Jonsson Comprehensive Cancer Center, Los Angeles, CA, USA; 4 Department of Psychiatry and Biobehavioral Sciences, David Geffen School of Medicine and Semel Institute, Los Angeles, CA, USA; 5 Department of Psychology, UCLA, Los Angeles, CA, USA; 6 Cousins Center for Psychoneuroimmunology, UCLA, Los Angeles, CA, USA; 7 Medical Oncology, Dana-Farber Cancer Institute, Boston, MA, USA; 8 The Johns Hopkins University School of Medicine and Sidney Kimmel Comprehensive Cancer Center, Baltimore, MD, USA; 9 Connors Center for Women's Health and Gender Biology, Brigham and Women's Hospital, and Harvard Medical School, Boston, MA, USA; 10 Department of Psychiatry, Brigham and Women’s Hospital, Boston, MA, USA; 11 Department of Psychosocial Oncology and Palliative Care, Dana-Farber Cancer Institute, Boston, MA, USA; 12 Department of Biostatistics, UCLA Fielding School of Public Health, Los Angeles, CA, USA

## Abstract

**Background:**

Major cancer organizations recommend depression screening in patients and survivors. The 9-item Patient Health Questionnaire (PHQ-9) is often suggested, with limited information about its use.

**Methods:**

Enrollment data collected from younger breast cancer survivors participating in a behavioral intervention trial were used to examine the relationship between PHQ-9 scores (range = 0-27), patient characteristics, and responses to standardized psychosocial assessment tools. Major depressive disorder criterion was met if responses to the first 2 PHQ-9 items (range = 0-6) were 3 or greater. The sample was categorized by total PHQ-9 scores: less than 5 (minimal depressive symptoms), 5-9 (mild to moderate depressive symptoms), and 10 or greater (moderate to severe depression). PHQ-9 category associations with medical, demographic, psychosocial, and behavioral characteristics were examined using analysis of variance for continuous variables and χ^2^ tests for categorical variables.

**Results:**

A total of 231 women met the study prescreening eligibility criterion of mild depressive symptoms and enrolled in the study. On average, they were 45.2 years old and 2.6 years since diagnosis. At enrollment, 22.1% met the screening criterion for possible major depressive disorder; among those with PHQ-9 scores of 10 or greater, 58.3% met this criterion. Anxiety, fatigue, insomnia, and intrusive thoughts about cancer were frequent and were associated with depressive symptom severity (all *P* < .001). In contrast, neither demographic nor cancer treatment characteristics were associated with depressive symptoms.

**Conclusions:**

Depressive symptoms in this selected sample of younger breast cancer survivors were independent of demographic characteristics or cancer treatment history, suggesting that depression screening is necessary to detect uncontrolled depressive symptoms.

Screening for depression in cancer patients is recommended in multiple professional guidelines and by accreditation bodies (1-3), as well as more generally for younger and middle-aged women, due to the high prevalence of major depressive disorder (MDD) (4). The 9-item Patient Health Questionnaire (PHQ-9) (5,6), a validated, widely used, brief questionnaire that assesses depressive symptoms and identifies individuals at risk for MDD (7), is recommended for screening in cancer patients (1). The PHQ-9 is thus a dual-purpose instrument that can suggest a provisional MDD diagnosis as well as grade depressive symptom severity (8). Clinical interventions are widely available for cancer patients with depressive symptoms; screening is the first step in identifying those in need of mental health evaluation (9,10). There are few published studies applying the PHQ-9 to cancer patients (11-13), with limited information about systematically screened cancer patients using this questionnaire (14).

Screening for depressive symptoms is particularly important for high-risk groups, including younger breast cancer survivors (YBCS). Breast cancer is the most common cancer in younger women (<50 years at diagnosis) (15,16), who make up approximately 19% of incident breast cancer cases (16). Most YBCS can expect several decades of survival after treatment ends because of advances in treatment. However, they are at high risk for adverse psychological and physical problems that can negatively affect the quality and length of survival (17–19). They are at elevated risk for breast cancer recurrence and cancer-specific mortality (15) as well as secondary cancers, premature menopause, infertility, osteoporosis, and cognitive decline (17,20–22). Younger women report more serious mental health issues and higher levels of depressive symptoms than older survivors, which may endure for years after successful cancer treatment (20,23). Psychological problems can interfere with adherence to medical recommendations, increasing morbidity and mortality, calling for ongoing assessment of depressive symptoms during the posttreatment survivorship period.

The Pathways to Wellness (PTW) study (Clinical Trials.gov NCT03025139) is a randomized clinical trial that tested 2 intervention programs designed to address elevated depressive symptoms in YBCS (24). Participants were prescreened for elevated depressive symptoms. At study enrollment, participants completed the PHQ-9 along with a comprehensive self-report questionnaire, including standardized assessments of symptoms, quality of life, and intrusive thoughts. We report on the relationship between the severity of PHQ-9 depressive symptoms and important patient characteristics, cancer treatments, and psychological and behavioral risk factors for depression.

## Methods

### Study Design

PTW is a multisite, randomized, 3-arm, phase III trial designed to evaluate the efficacy of 2 distinct group interventions (mindfulness meditation; survivor education) compared with a wait-list control group for reducing depressive symptoms. The study was approved by the institutional review boards of each participating institution.

### Study Eligibility

Inclusion criteria were as follows: 1) breast cancer diagnosis (stage 0, I, II, or III) at or before age 50 years; 2) within 5 years of diagnosis; 3) completion of surgery, radiation, and/or chemotherapy at least 6 months previously; 4) ability to complete study questionnaires in English; 5) ability to participate in the 6-week group intervention; and 6) presence of at least mild depressive symptoms (see below for details). Exclusion criteria were: 1) recurrent or metastatic breast cancer; 2) another interval cancer diagnosis following breast cancer (excluding nonmelanoma skin cancer); 3) current mindfulness practice; 4) pregnancy; and 5) chronic medical or psychiatric condition that could limit participating in the intervention programs or interpretation of the trial outcome assessment.

### Procedures

Study recruitment occurred at 3 institutions: UCLA Jonsson Comprehensive Cancer Center in Los Angeles, CA; Dana-Farber Cancer Institute in Boston, MA; and Johns Hopkins Kimmel Cancer Center in Baltimore, MD. Recruitment materials described a study of 2 separate 6-week group wellness programs specifically designed for YBCS. Institutional and community resources were used to identify potentially eligible study participants, including regional cancer registries. Recruitment procedures included mailed invitation letters, electronic mailings, flyers, and social media announcements, as well as direct invitation by treating clinicians.

YBCS were screened by telephone to determine interest and eligibility, and for those agreeing to participate, an enrollment in-person visit was scheduled. Written informed consent was obtained at that visit, and baseline study assessments were conducted. The enrollment electronic Research Electronic Data Capture (REDCap) questionnaire asked about demographic, medical, and mental health history (history of depressive episode and medication treatment); health behaviors (tobacco and alcohol use); breast cancer treatment history; work history; comorbid conditions and medications; and standardized psychosocial and behavioral questionnaires.

### Depressive Symptoms at Screening and Study Enrollment

The PHQ-8 item (PHQ-8) (25) was administered during eligibility telephone screening. The PHQ-8 excludes the ninth item assessing suicidality (see PHQ-9 items listed in Box 1). To be eligible for PTW, screening scores of 5 or greater were required, which is the threshold score for mild depressive symptoms (5). PTW study participants completed the full PHQ-9 (range = 0-27; see Box 1) at the enrollment visit. The PHQ-9 is the depression module of the PRIME MD diagnostic instrument and scores each of the 9 *DSM-IV* criteria for MDD as 0 (not at all) to 3 (nearly every day) over the past 2 weeks. The 9 items were depressed mood, anhedonia (little interest or pleasure), disturbed sleep, fatigue, eating too much or too little, trouble concentrating, psychomotor retardation or agitation, low self-worth, and suicidal ideation. If the suicidal ideation item was endorsed, research staff conducted a standardized interview to evaluate suicidal intent that was reviewed with a licensed clinician. Criterion for risk of MDD was met if responses to the first 2 items of the PHQ-9 (PHQ-2 items, range = 0-6) were 3 or greater; these items assess the 2 cardinal symptoms of depression: depressed mood and anhedonia (7,26). The full PHQ-9 was used to categorize the sample on depressive symptom severity: score less than 5 (minimal depressive symptoms), 5-9 (mild to moderate depressive symptoms), and 10 or greater (moderate to severe depression), which are validated scale cut points based on standardized thresholds (5,8).

Box 1 Patient Health Questionnaire (PHQ-9)

Over the last 2 weeks, how often have you been bothered by any of the following problems?

Responses: not at all (0), several days (1), more than half the days (2), nearly every day (3)

1. Little interest or pleasure in doing things
2. Feeling down, depressed, or hopeless
3. Trouble falling or staying asleep or sleeping too much
4. Feeling tired or having little energy
5. Poor appetite or overeating
6. Feeling bad about yourself—or that you are a failure or have let yourself or your family down
7. Trouble concentrating on things, such as reading the newspaper or watching television
8. Moving or speaking so slowly that other people could have noticed or the opposite—being fidgety or restless that you have been moving around a lot more than usual
9. Thoughts that you would be better off dead, or of hurting yourself in some way

### Other Study Measures

Fatigue was assessed with the Fatigue Symptom Inventory (FSI), a 13-item measure that assesses fatigue intensity, duration, and interference with daily functioning (27). We focused on fatigue severity (assessed with 4 items that capture most fatigue, least fatigue, average fatigue, and current fatigue) and fatigue duration (assessed with 1 item). Subjective sleep quality and insomnia symptoms were assessed with the Insomnia Severity Index (ISI), which has 7 items that are summed with a range of 0-28 (28). Side effects of cancer treatment were assessed with 20 items from the Breast Cancer Prevention Trial (BCPT) Symptom Scales, which include a variety of physical symptom scales relevant to breast cancer survivors and are scored from 0 to 4 in severity (29). The Short Form Health Survey 12 item (SF*-*12) health survey (30) was used to capture the broad domains of physical and mental health functioning at each assessment. We used norm-based scoring, with a score of 50 representing the population mean, and 10 points higher or lower indicating 1 SD. Effects of cancer on work and other activities were assessed with the 6-item Work Productivity and Activity Impairment Questionnaire, with scores for each item ranging from 0 to 10: 0 = no impact of health on work, to 10 = health problems completely preventing ability to work (31). Clinically significant anxiety was assessed using the Generalized Anxiety Disorder-7 (GAD-7) questionnaire, a 7-item scale that assesses anxiety symptoms in the past 2 weeks (32). Cancer-related distress was assessed with the intrusions subscale of the Impact of Events Scale (IES) (33), focused on cancer. Mean and range of scores as well as clinical cut points for relevant scales are presented in tables associated with results.

### Statistical Analysis

Participants were categorized by PHQ-9 scores as described above, and associations of category with medical, demographic, psychosocial, and behavioral characteristics were assessed using analysis of variance for continuous variables and χ^2^ tests for categorical variables. SAS version 9.4 (SAS Institute, Cary, NC) was used for all analyses. Because this was an exploratory evaluation of associations with the PHQ-9 categories, no correction was made for multiple comparisons. All statistical tests were 2-sided, and a *P* value of less than .05 was considered statistically significant.

## Results

### Recruitment of the Study Sample

Eligibility screening and enrollment were conducted between fall 2017 and fall 2019. Screening occurred a median of 40 days before enrollment, with an interquartile range of 22-66 days. There were 1525 women who initially expressed an interest in the study, but only 1216 agreed to formal screening. Among the latter, 532 were not interested and 433 were deemed ineligible; 242 of the 433 (55.9%) were ineligible because of a PHQ-8 score that was too low. Ultimately, 251 were enrolled and randomly assigned. Of these 251, four were excluded from the baseline sample (3 found medically ineligible after random assignment, and 1 with unevaluable data), and 16 were excluded from this analysis due to a screening PHQ-8 score of 3 or 4, leaving a final sample of 231 for this analysis who had a screening PHQ-8 score of 5 or greater.

### Sample Characteristics by PHQ-9 Score Category

The mean PHQ-9 score at study enrollment was 8.0 (SD = 4.8; range = 0-26). One-quarter (22.9%) of participants scored below 5 on the PHQ-9 (despite having a screening score >5), 45.9% scored between 5 and 9, and 31.2% scored 10 or greater. Table 1 shows demographic and medical characteristics of the study sample by PHQ-9 categories. Participants were 45.2 years (SD = 6.4) on average and were 2.6 years (SD = 1.1) since diagnosis. The sample was 81.2% White, 8.3% Black, 8.3% Asian, and 2.2% other; 10.0% were Hispanic. The majority were married or in a partnered relationship and well-educated with higher income status. Sixty percent (n = 139) had children. Only age was statistically significantly different among the 3 PHQ-9 groups (*P* = .02), with those with a PHQ-9 score less than 5 being the oldest. There were no statistically significant differences among the groups for race, ethnicity, marital status, education, employment, household income, or having children.

**Table 1.** Demographic and medical characteristics by PHQ-9 category score

Characteristic	PHQ-9 category score			All women	*P*^a^
	<5	5-9	10+		
Total No.	53 (22.9)	106 (45.9)	72 (31.2)	231 (100)	
Demographics					
Site, No. (%)					
Dana Farber	16 (30.2)	49 (46.2)	23 (31.9)	88 (38.1)	.23
Johns Hopkins	13 (24.5)	20 (18.9)	16 (22.2)	49 (21.2)	
UCLA	24 (45.3)	37 (34.9)	33 (45.8)	94 (40.7)	
Current age, mean (SD) [range], y	47.4 (4.6) [34.9-55.4]	44.4 (6.6) [23.2-54.5]	44.8 (7.1) [26.5-54.2]	45.2 (6.4) [23.2-55.4]	.02
Age at diagnosis, mean (SD), y	44.7 (4.2)	42.0 (6.2)	42.1 (7.0)	42.6 (6.2)	.02
Years from diagnosis, mean (SD)	2.7 (1.2)	2.5 (1.1)	2.7 (1.1)	2.6 (1.1)	.40
Race, No. (%)					
White (includes Hispanic)	44 (83.0)	83 (79.1)	59 (83.1)	186 (81.2)	.74^b^
Black, non-Hispanic	6 (11.3)	8 (7.6)	5 (7.0)	19 (8.3)	
Asian	2 (3.8)	10 (9.5)	7 (9.9)	19 (8.3)	
Other	1 (1.9)	4 (3.8)	0 (0.0)	5 (2.2)	
Hispanic, No. (%)	7 (13.2)	12 (11.3)	4 (5.6)	23 (10.0)	.30
Marital status, No. (%)					
Married or living as married	35 (66.0)	71 (67.0)	42 (58.3)	148 (64.1)	.47^c^
Divorced	4 (7.6)	18 (17.0)	14 (19.4)	36 (15.6)	
Widowed	0 (0.0)	1 (0.9)	1 (1.4)	2 (0.9)	
Single	14 (26.4)	16 (15.1)	15 (20.8)	45 (19.5)	
Education, No. (%)					
No college degree	11 (20.8)	21 (19.8)	11 (15.3)	43 (18.6)	.88
College degree	24 (45.3)	45 (42.5)	31 (43.1)	100 (43.3)	
Postgraduate degree	18 (34.0)	40 (37.7)	30 (41.7)	88 (38.1)	
Employment status, No. (%)					
Employed full-time	34 (64.2)	76 (71.7)	46 (63.9)	156 (67.5)	.52
Employed part-time	9 (17.0)	11 (10.4)	14 (19.4)	34 (14.7)	
Not employed	10 (18.9)	19 (17.9)	12 (16.7)	41 (17.8)	
Annual household income (missing = 17), No. (%)					
<$60 K	11 (22.5)	17 (17.4)	17 (25.4)	45 (21.0)	.46
$60K-$100K	10 (20.4)	20 (20.4)	18 (26.9)	48 (22.4)	
>$100 K	28 (57.1)	61 (62.2)	32 (47.8)	121 (56.5)	
Medical characteristics					
BMI, mean (SD), kg/m^2^	27.4 (6.7)	26.1 (5.6)	27.8 (6.2)	26.9 (6.0)	.16
Surgery, No. (%)					
Lumpectomy	18 (34.0)	41 (38.7)	33 (45.3)	92 (39.8)	.24
Mastectomy	35 (66.0)	59 (55.7)	36 (50.0)	130 (56.3)	
No surgery	0 (0.0)	6 (5.7)	3 (4.2)	9 (3.9)	
Had chemotherapy, No. (%)	30 (56.6)	70 (66.0)	43 (59.7)	143 (61.9)	.46
Had radiation, No. (%)	31 (58.5)	70 (66.0)	50 (69.4)	151 (65.4)	.44
Took trastuzumab (missing = 5), No. (%)	9 (17.0)	32 (30.8)	17 (24.6)	58 (25.7)	.17
Endocrine therapy, No. (%)					
Current	32 (60.4)	68 (64.2)	51 (70.8)	151 (64)	.45
Past (missing = 6)	6 (11.5)	17 (16.5)	12 (17.1)	35 (15.6)	.66
Ovarian suppression, Current, No. (%)	8 (15.1)	17 (16.0)	9 (12.5)	34 (14.7)	.80
Menopausal status at diagnosis, No. (%)					
Premenopausal	34 (64.2)	78 (74.3)	57 (80.3)	169 (73.8)	.13^d^
Perimenopausal	7 (13.2)	7 (6.7)	9 (12.7)	23 (10.0)	
Postmenopausal	4 (7.6)	1 (1.0)	1 (1.4)	6 (2.6)	
Unknown	8 (15.1)	19 (18.1)	4 (5.6)	31 (13.5)	

a Two-sided *P* values less than .05 were considered statistically significant; analyses used analysis of variance for continuous variables and χ^2^ tests for categorical variables. BMI = body mass index; PHQ-2 = Patient Health Questionnaire 2 item; PHQ-9 = Patient Health Questionnaire 9 item; UCLA = University of California Los Angeles.

b White vs non-White.

c Married vs unmarried.

d Premenopausal vs other.

There were no statistically significant differences in medical variables among the 3 groups (Table 1), with the majority having received mastectomy (56.3%), chemotherapy (61.9%), radiation (65.4%), and current endocrine therapy (64%); 15.6% reported past receipt of endocrine therapy and 14.7% reported current receipt of ovarian suppression therapy. A total of 25.7% received HER2-directed therapy.

### Depression, Anxiety, and Intrusive Thoughts

Table 2 shows the association of standardized self-report assessments and depression-related interventions with the PHQ-9 categories. We first consider the PHQ-9 categories and the PHQ-2 scores, then history of depression, and current depression treatments. Based on their PHQ-2 scores, 22.1% met the criteria for risk of MDD, with statistically significant differences across the PHQ-9 groups (*P* < .001). Women with PHQ-9 scores 10 or greater were more likely to be classified at risk for MDD based on the PHQ-2 (58.3%). Additionally, 55.2% of the entire sample reported a past history of being depressed for 2 weeks or more, with statistically significant differences across the PHQ-9 categories (*P* = .004); those with PHQ-9 scores 10 or greater were the most likely to report a previous depression history (69.4%). However, only 24.4% of the full samples were currently taking medication for depression, with no difference across the PHQ-9 groups. Only 17.9% reported current receipt of psychotherapy or counseling, with no statistically significant differences in relationship to PHQ-9 scores.

**Table 2.** Assessment of depression, anxiety, intrusive thoughts, and treatments

Assessment instrument or questionnaire	PHQ-9 category score			All women (n = 231)	*P*^a^
	<5	5-9	10+		
	(n = 53)	(n = 106)	(n = 72)		
PHQ-9: possible range 0-27, mean (SD)	2.7 (1.2)	6.8 (1.3)	13.6 (4.1)	8.0 (4.8)	<.001
PHQ-2: possible range 0-6, mean (SD)	0.4 (0.6)	1.3 (1.0)	3.0 (1.5)	1.6 (1.5)	<.001
Binary PHQ-2, No. (%)					
0-2 (not depressed)	53 (100.0)	97 (91.5)	30 (41.7)	180 (77.9)	<.001
3-6 (depressed)	0 (0.0)	9 (8.5)	42 (58.3)	51 (22.1)	
PHQ-9 Item 9: No. (%)					
0. Not at all	53 (100.0)	101 (95.3)	55 (76.4)	209 (90.5)	<.001^b^
1. Several days	0 (0.0)	4 (3.8)	12 (16.7)	16 (6.9)	
2. More than half the days	0 (0.0)	1 (0.9)	4 (5.6)	5 (2.2)	
3. Nearly every day	0 (0.0)	0 (0.0)	1 (1.4)	1 (0.4)	
History of depression: ever been depressed for 2 wk or more? No. (%)	21 (39.6)	56 (53.3)	50 (69.4)	127 (55.2)	.004
Currently taking depression medication, No. (%)	12 (22.6)	20 (19.1)	24 (33.3)	56 (24.4)	.09
Individual psychotherapy/counseling for cancer-related problems or concerns, No. (%)					
Currently	6 (11.3)	19 (18.1)	16 (22.5)	41 (17.9)	.27^c^
In the past	9 (17.0)	21 (20.0)	19 (26.8)	49 (21.4)	
Never	38 (71.7)	65 (61.9)	36 (50.7)	139 (60.7)	
GAD-7, possible range 0-21, mean (SD)	5.2 (4.2)	6.9 (3.7)	10.2 (5.1)	7.5 (4.7)	<.001
Categorical GAD-7, No. (%)					
No anxiety (0-4)	27 (50.9)	22 (20.8)	10 (13.9)	59 (25.5)	<.001
Mild anxiety (5-9)	20 (37.7)	62 (58.5)	25 (34.7)	107 (46.3)	
Moderate anxiety (10-14)	4 (7.6)	18 (17.0)	22 (30.6)	44 (19.1)	
Severe anxiety (15-21)	2 (3.8)	4 (3.8)	15 (20.8)	21 (9.1)	
Currently taking anxiety medication, No. (%)	15 (28.3)	28 (26.4)	22 (30.6)	65 (28.1)	.83
IES Intrusions subscale—possible range 0-35, mean (SD)	8.5 (5.7)	13.3 (8.6)	16.4 (9.7)	13.1 (8.9)	<.001

a Two-sided *P* values less than .05 were considered statistically significant; analyses used analysis of variance for continuous variables and χ^2^ tests for categorical variables. GAD-7 = Generalized Anxiety Disorder 7-Item Scale, higher score indicates greater anxiety; IES = Impact of Events Scale, higher score indicates greater intrusive thoughts; PHQ-2 = Patient Health Questionnaire 2 item, higher score indicates greater depressive symptoms; PHQ-9 = Patient Health Questionnaire 9 item, higher score indicates greater depressive symptoms.

b *P* for 0 vs 1, 2, 3.

c *P* for currently vs not.

Another indicator of more severe depressive symptoms is suicidal ideation, assessed by the ninth item of the PHQ-9, which asks about “thoughts that you would be better off dead or of hurting yourself in some way.” Of the total sample, 9.5% (n = 22) endorsed this item, with the highest rates (23.7%) seen among those with a PHQ-9 score 10 or greater. Affirmative responses to this item were monitored and addressed as a safety procedure in the study conduct.

Anxiety, measured with the GAD-7, was prevalent in these YBCS and was strongly associated with increasing scores on the PHQ-9 (*P* < .001); indeed, 51.4% of women with a PHQ-9 score 10 or greater met the criteria for clinically significant anxiety symptoms. As with antidepressant medications, few women were currently taking medication for anxiety. Intrusive thoughts about cancer, measured by the IES, were frequent, with higher severity statistically significantly associated with higher PHQ-9 scores (*P* < .001).

### Health-Related Quality of Life and Behavioral Symptoms

Table 3 provides data on quality of life, behavioral symptoms, and other problems by PHQ-9 category. The overall SF-12 Physical Component Scale (PCS) score was at the population norm (30) for those with PHQ-9 scores less than 10 but was about 0.5 SD lower in those with a score of 10 or greater (*P* = .003). Consistent with expectations, the mean SF-12 Mental Component Scale (MCS) score was statistically significantly lower in those with higher PHQ-9 scores (*P* < .001), and remarkably, those with a PHQ-9 score 10 or greater had a SF-12 MCSscore of 31.5, which is nearly 2 SD below the population normative score of 50, indicating serious impairment in mental health functioning. Using standard measures for fatigue and insomnia, 97.2% of those with PHQ-9 scores 10 or greater met the criteria for clinically significant fatigue (FSI ≥ 3), and 58.4% met the criteria for clinically moderate or severe insomnia (ISI ≥ 15).

**Table 3.** Quality of life, symptoms, and behavioral problems

Questionnaire	PHQ-9 category score			All women (n = 231)	*P*^a^
	<5	5-9	10+		
	(n = 53)	(n = 106)	(n = 72)		
SF-12 version 1: mean (SD)					
PCS	51.6 (7.9)	51.0 (8.6)	46.3 (11.9)	49.6 (9.9)	.003
MCS	47.0 (8.6)	41.6 (8.3)	31.5 (9.3)	39.6 (10.5)	<.001
FSI severity, possible range 0-10, mean (SD)	3.3 (1.5)	4.5 (1.4)	5.6 (1.5)	4.6 (1.6)	<.001
Binary FSI severity, No. (%)					
<3 (not fatigued)	21 (39.6)	13 (12.3)	2 (2.8)	36 (15.6)	<.001
3 or more (fatigued)	32 (60.4)	93 (87.7)	70 (97.2)	195 (84.4)	
ISI, possible range 0-28, mean (SD)	8.4 (4.5)	12.1 (5.6)	15.0 (6.9)	12.1 (6.3)	<.001
Categorical ISI, No. (%)					
No clinically significant insomnia (0-7)	24 (45.3)	25 (23.6)	12 (16.7)	61 (26.4)	<.001
Subthreshold insomnia (8-14)	24 (45.3)	46 (43.4)	18 (25.0)	88 (38.1)	
Clinical insomnia, moderate severity (15-21)	4 (7.6)	30 (28.3)	31 (43.1)	65 (28.1)	
Clinical insomnia, severe (22-28)	1 (1.9)	5 (4.7)	11 (15.3)	17 (7.4)	
BCPT Symptom Scales, possible range 0-4, mean (SD)					
Hot flashes	1.2 (1.2)	1.4 (1.2)	1.8 (1.5)	1.5 (1.3)	.03
Nausea	0.1 (0.2)	0.2 (0.4)	0.3 (0.5)	0.2 (0.4)	.02
Bladder control	0.2 (0.3)	0.5 (0.8)	0.8 (1.2)	0.5 (0.9)	.006
Vaginal problems	0.6 (1.0)	0.9 (1.2)	1.1 (1.3)	0.9 (1.2)	.12
Musculoskeletal pain	1.2 (1.0)	1.4 (1.1)	2.0 (1.2)	1.6 (1.1)	<.001
Cognitive problems	1.0 (0.8)	1.6 (0.9)	2.6 (1.1)	1.8 (1.1)	<.001
Weight problems	1.1 (1.0)	1.9 (1.2)	2.4 (1.4)	1.9 (1.3)	<.001
Arm problems	0.6 (0.9)	0.6 (0.8)	1.0 (1.2)	0.7 (1.0)	.01

a Two-sided *P*s less than .05 were considered statistically significant; analyses used analysis of variance for continuous variables and chi-square tests for categorical variables. BCPT = Breast Cancer Prevention Trial, higher score indicates greater severity of symptom; FSI = Fatigue Severity Inventory, higher score indicates more fatigue; ISI = Insomnia Severity Index, higher score indicates greater insomnia; MCS = mental component scale; PCS = physical component scale; SF-12 = Short Form Health Survey 12 item, higher is better.

Greater severity of depressive symptoms was associated with statistically significantly more severe breast cancer treatment–related symptoms (eg, musculoskeletal pain, cognitive difficulties, weight concerns, and arm problems) shown in Table 3. Employment rates were similar across the groups, with 81% employed; however, women with higher PHQ-9 scores reported statistically significantly greater impact of their health on work productivity (*P* < .001) and how much their health problems affected their ability to do regular activities other than work (*P* < .001) (data not shown).

### Other Behaviors

There were no differences across the 3 groups in smoking or alcohol use, with only 5% self-reporting being current smokers and a mean self-report consumption of alcohol in the past week of 2.2 (SD = 2.8) drinks of any kind of alcoholic beverage. Participants were queried about current use of integrative medicine practices (meditation, yoga, Tai Chi, or Qigong) or participation in a cancer support group. No statistically significant differences in the use of these services in relationship to the severity of depressive symptoms were noted (data not shown).

## Discussion

The PHQ-9 (or shorter versions) is widely used in primary care practice for clinical depression screening (5,6,25); however, its use in clinical oncology populations has been limited (11–14). Most oncology studies have focused on the diagnostic accuracy of the PHQ-9 in screening for MDD rather than characterizing a population of cancer patients who have PHQ-9–identified depressive symptoms (11,12). In this selected sample of patients participating in a behavioral intervention trial, depressive symptom severity was not associated with any demographic characteristics (eg, education, marital status) or cancer treatments (eg, chemotherapy, type of surgery) received in the past or that were ongoing, such as endocrine therapy. This has been previously reported in breast cancer patients (34,35), suggesting that standardly available demographic and medical characteristics should not be used to select who should be screened for depression.

The current sample was prescreened for presence of at least mild depressive symptoms, and almost one-quarter (22%) met the criterion for risk of MDD, as indicated by endorsement of depressed mood and/or anhedonia in the past 2 weeks. For those with PHQ-9 scores 10 or greater, the MDD rate was 58%. Lack of treatment for current depressive symptoms in this patient sample is concerning, with low reported use of medications or counseling among those with high levels of distress, although patients may have been offered and declined mental health referrals or medication prescriptions. Nevertheless, these are noteworthy observations considering that the majority of YBCS were recruited from 3 urban NCI-funded comprehensive cancer centers with access to enriched institutional psychosocial resources.

An alarming finding was the high suicidal ideation rate among the PTW participants—10% overall, with a proportionately greater rate in those with PHQ-9 scores 10 or greater (24%), and no endorsement among those with scores less than 5. This high prevalence of suicidal thoughts required active assessment of suicidal intent as part of the research protocol and would also be required if the PHQ-9 is used as a screening tool in clinical practice. Suicide is strongly associated with uncontrolled depressive symptoms (36), and the literature has identified YBCS as a high-risk group for suicide (37,38). Increased anxiety was also prevalent among this patient sample and statistically significantly associated with increasing PHQ-9 scores. As with depression, medication for anxiety was only reported in approximately one-quarter of the patients and was not related to the severity of symptoms. Finally, those with a PHQ-9 score 10 or greater also suffered from greater intrusive thoughts, which has been linked to posttraumatic stress disorder (39).

Independent of depression, fatigue, anxiety, and insomnia are burdensome for these YBCS, and there are many successful management options for control of these symptoms. YBCS with even mild levels of depressive symptoms (PHQ-9 scores of 5-9) had substantial fatigue and insomnia, which if left unaddressed could exacerbate depressive symptoms. PHQ-9 screening identifies several important targets for behavioral or pharmacological intervention, independent of its value in screening for MDD, and can be seen as providing “added value” when used as a tool for clinical depression screening. Among the women who were working, those with higher PHQ-9 scores reported statistically significantly greater impact of their health on work productivity and that their health problems affected their ability to do regular daily activities other than work, which is supported by the statistically significant association of fatigue, insomnia, and anxiety with greater levels of depressive symptoms.

An important limitation is the selected nature of this patient sample, who were participants in a research study and were initially screened for depressive symptoms during study recruitment. Of note, initial depression screening occurred a median of 40 days before enrollment, and by the time the PHQ-9 was completed, 23% no longer reported elevated depressive symptoms (>5). Study participants had to be willing to enter a randomized trial to receive a “wellness intervention,” accepting assignment to attend an in-person, group program over a 6-week period and to come to one of the sites for the baseline visit before random assignment (40). All of these factors likely increased the selectivity of the sample. However, we did not suggest to potential participants that this was a therapeutic intervention program, such as a support group, and the interventions offered followed a curriculum that focused on either mindfulness training or education about breast cancer survivorship.

Depression is responsible for decreased quality of life, reduced productivity, and mortality and substantial disability, and globally is a major challenge at the population level (41,42). Depression is much more prevalent in younger adults aged younger than 50 years, with 7%-9% reporting a history of MDD (43) and adult women overall having a past-year MDD prevalence of 8.7% compared with 5.3% in men (44). Depressed individuals often clinically report somatic symptoms rather than symptoms of sadness or altered mood, making screening for depression an opportunity to identify those in need of further evaluation and intervention. As cancer patients move beyond the acute phase of treatment, less attention is paid to their psychosocial needs (45), yet often this is a time of greater distress for patients because they must learn to live with the aftereffects of treatment and the “new normal.” A recent prospective study of early-stage breast cancer patients followed out to 3 years postdiagnosis confirmed worsening symptoms (depression, anxiety, fatigue) over time, especially in premenopausal women (46). Given these findings and our data, screening for depression in YBCS should continue at regular intervals during the posttreatment survivorship period.

Recommendations of the Institute of Medicine (47), the American Society of Clinical Oncology (1), the Commission on Cancer (3), and Center for Medicare and Medicaid Services Oncology Care Model (48) all include screening for depression as a quality metric in cancer patients and survivors. Depression is often treatable with pharmacologic and nonpharmacologic interventions, and screening for depression is designed to help reduce suffering and improve well-being among those living beyond cancer (49). Adverse mental health outcomes, such as anxiety, depression, and suicide, are increased in YBCS compared with women without a cancer history, emphasizing the need for screening, prevention, and management strategies (50). In this research study, we identified a particularly vulnerable group of YBCS merely by screening for study eligibility using a standardized depression screening tool to detect those with at least a mild level of depressive symptoms. In a comprehensive review of depressive spectrum disorders in cancer, Caruso et al. (51) note “survivorship is not exempt from mood disorders and given the large number of patients in the survivorship phase the overall burden of depression should also be considered in this phase. This highlights the need to avoid abandoning long survivors even if in remission as many will still suffer from depressive spectrum disorders.” 

## Funding

This research was supported by NCI R01 CA200977 and NCI P30 CA016042 as well as by NIH National Center for Advancing Translational Science (NCATS) UCLA CTSI Grant Number UL1TR001881. Additional support was provided by the Breast Cancer Research Foundation (BCRF).

## Notes

**Role of the funders:** The funding sources played no role in the design, execution of the research, writing of the manuscript, or decision to submit the manuscript for publication.

**Acknowledgements:** The authors thank the Pathways to Wellness Study participants, as well as the research staff at all of our institutions who facilitated the recruitment and enrollment of patients into the study.

**Disclosures:** PAG is a member of the BCRF Scientific Advisory Board, a funder of the study. The other authors have no disclosures.

**Author contributions:** Conceptualization and design-PAG, JEB, CMC. Data acquisition: PAG. JEB, AHP, ACW, EDT. Data analysis and interpretation: PAG, JEB, LP, CMC. Manuscript drafting and editing: PAG, JEB, LP, CMC. Manuscript review, editing and final approval: PAG, JEB, AHP, ACW, EDT, HJ, MRI, LP, CMC.

## Data Availability

The data underlying this article will be shared on reasonable request to the corresponding author.
